# Impact of hyperprolactinemia in a patient with polyautoimmunity

**DOI:** 10.1002/ccr3.1900

**Published:** 2018-11-09

**Authors:** Manuel Rojas, Yhojan Rodríguez, Carolina Ramírez‐Santana, Juan‐Manuel Anaya

**Affiliations:** ^1^ Center for Autoimmune Diseases Research (CREA), School of Medicine and Health Sciences Universidad del Rosario Bogota Colombia; ^2^ Doctoral Program in Biomedical Sciences Universidad del Rosario Bogotá Colombia

**Keywords:** hyperprolactinemia, prolactin, Sjögren's syndrome, systemic lupus erythematosus

## Abstract

Hyperprolactinemia has been proposed as a triggering factor for autoimmune diseases. The increased levels of prolactin could induce an abnormal immune response. Herein, we present a patient with hyperprolactinemia who developed polyautoimmunity. Patient's symptoms were associated with slightly raised levels of prolactin (20‐40 ng/mL) and administration of dopaminergic agonists.

## 1 BACKGROUND

The role of prolactin (PRL) in the development of autoimmune diseases (ADs), such as systemic lupus erythematosus (SLE) and rheumatoid arthritis (RA), has been previously recognized.^1^ Consequently, hyperprolactinemia has been postulated as a triggering factor of autoimmunity and as a predictor of disease activity and severity.^1^ PRL is related to a wide variety of functions including mammary gland growth, lactation, stress response, immune system development, and autoimmunity.^1^ In fact, it was recognized that at high PRL levels (>100 ng/mL) the immune system is inactivated; however, sometimes at slightly raised levels (20‐40 ng/mL) the immune activity increases.^2^ In this case report, we described a patient with hyperprolactinemia and polyautoimmunity (PolyA), who had lupus flares with PRL levels between 20 and 40 ng/mL, and presented remission of symptoms with PRL concentrations above 100 ng/mL.

## 2 CASE REPORT

**Figure 1:**
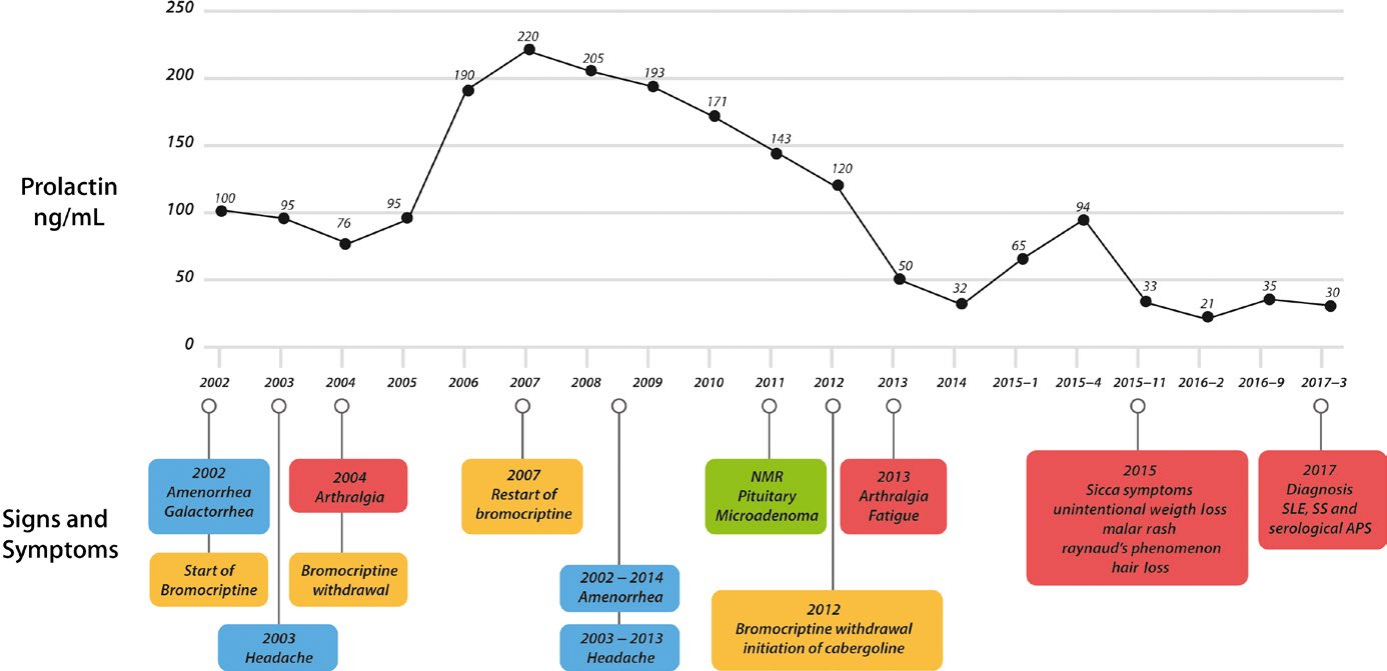
Clinical timeline. Prolactin concentrations are shown through the last 15 years. Blue boxes represent hyperprolactinemia‐related symptoms. Red boxes represent autoimmune manifestations. Yellow boxes show treatments. Green box indicates the time NMR was done. APS, antiphospholipid syndrome; NMR, nuclear magnetic resonance; SLE, systemic lupus erythematosus; SS, Sjögren's syndrome

**Figure 2:**
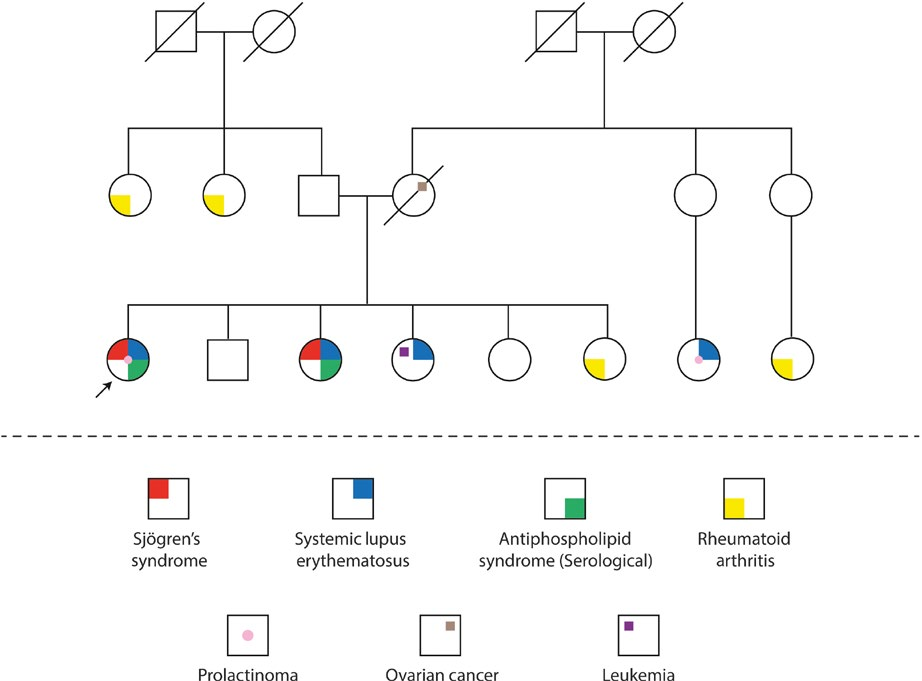
Proband pedigree. Specific autoimmune diseases are in colors. The pedigree discloses both polyautoimmunity (ie, two or more autoimmune diseases in a single patient) and familial autoimmunity (ie, diverse autoimmune diseases in a nuclear family). One patient's cousin suffers from prolactinoma and SLE

We present a 36‐year‐old woman with 1‐year history of Raynaud's phenomenon, pain, and paresthesia, without clinical signs or symptoms of ischemia or vasculitis. Additionally, she complained of morning pain and stiffness involving interphalangeal and metacarpophalangeal joints, malar rash, subjective hair loss, mouth ulcers, weight loss of 5 kg in the last 3 months, and *sicca* symptoms. She denied visual disturbances, headache, dizziness, galactorrhea, or amenorrhea at the time of consultation. This clinical picture was associated with PRL levels between 20 and 40 ng/mL (Figure 1), despite cabergoline treatment at 0.5 mg twice per week. Interestingly, she presented with familial autoimmunity and one relative with SLE associated with prolactinoma (Figure 2).

Hyperprolactinemia was diagnosed in 2002 due to secondary amenorrhea; hence, bromocriptine was started at 1.25 mg per day. No musculoskeletal symptoms were present at the moment of diagnosis. After 2 years with bromocriptine, PRL levels decreased at 72 ng/mL, but at the same time, she developed morning stiffness and arthralgia. Due to symptoms recurrence, she decided to withdraw bromocriptine for 3 years with paradoxical improvement of her musculoskeletal symptoms. In 2007, amenorrhea recurred due to high levels of PRL; therefore, bromocriptine was restarted (Figure 1).

After 5 years of treatment, high levels of PRL persisted despite bromocriptine prescription. A magnetic resonance image was performed, disclosing a pituitary microadenoma. Consequently, bromocriptine was changed for cabergoline 0.5 mg twice per week. This treatment showed an evident decrease in PRL concentration. Interestingly, after treatment adjustment, arthralgia, fatigue, and malar rash returned. In 2015, when lower PRL levels were reached, new onset of Raynaud's phenomenon, *sicca* symptoms, alopecia, and unintentional weight loss were identified (Figure 1). At that time, mild leuko‐lymphopenia (leukocytes: 4.000/mm^3^, lymphocytes 1.400/mm^3^) was observed, and serological tests for infectious diseases (including syphilis, toxoplasmosis, rubella, and HIV) were negative. Renal and hormonal function tests, including thyroid and hypothalamic‐pituitary‐gonadal axis, were normal.

**Figure 3:**
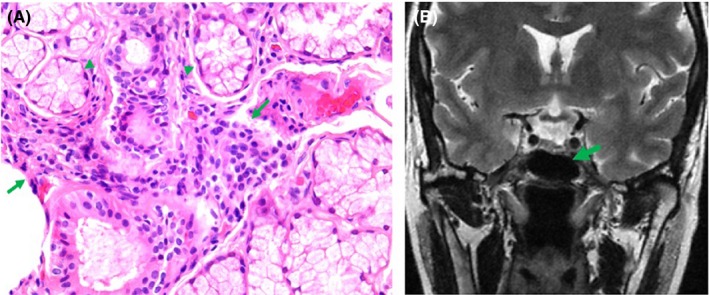
(A) Minor salivary gland biopsy, 40×. Arrows: lymphocytic infiltration. Arrowheads: Salivary acinus. (B) Sella turcica nuclear magnetic resonance. Green arrow: asymmetry of pituitary gland due to pharmacology treatment with cabergoline and bromocriptine

Blood tests from January 2017 showed mild anemia (11.6 g/dL), peripheral smear with poikilocytosis, ovalocytes, and target cells, and normal renal function. Immunologic tests were positive for ANAs 1:160 (speckled pattern), dsDNA, anti‐Sm, anti‐SS‐A/Ro, anti‐SS‐B/La, anti‐RNP autoantibodies, lupus anticoagulant (LA), and anticardiolipin antibodies (ACA) III IgM and IgG, but negative for rheumatoid factor, anticitrullinated peptide antibodies, antithyroid peroxidase, antithyroglobulin, and β2GP1 antibodies. The minor salivary gland biopsy showed a focus score >1 (Figure 3A), and the sella turcica nuclear magnetic resonance (STNMR) disclosed pituitary asymmetry (Figure 3B). She carried HLA‐A*01:01:01G/A*24:02:01G, HLA‐B*45:01:01G/B*57:03:01G, HLA‐C*16:01:01G/C*18:01:01G, HLADRB1*10:01:01G/DRB1*15:03:01G, and HLA‐DQB1*05:01:01G/DQB1*06:02:01G.

A diagnosis of PolyA characterized by the presence of SLE and Sjögren's syndrome (SS) was made. Treatment with hydroxychloroquine (200 mg/d), deflazacort (6 mg/d), and acetylsalicylic acid (100 mg/d) was started. Cabergoline was continued (0.5 mg twice per week). One year after follow‐up, signs and symptoms improved, including malar rash, arthralgia, and Raynaud's phenomenon associated with levels of PRL below 20 ng/mL.

## 3 DISCUSSION

PRL receptor belongs to the family of cytokine receptors associated with interleukin IL‐2, IL‐6, granulocyte‐macrophage colony‐stimulating factor (GM‐CSF), and leptine.^3^ These receptors are linked to secondary messengers such as JAK/STAT and MAPK, which act through NF‐κB, enhancing proliferative response to antigens, increasing production of cytokines and autoantibodies.^4^ Thus, the Th1 (cellular immunity) or Th2 (humoral immunity) cell responses are induced in presence of PRL.^5^

Our patient illustrates the paradoxical effect of PRL levels on the development of autoimmunity, as was observed by Jacobi et al^2^ who described that in peripheral mononuclear cells from patients with SLE, concentrations of PRL at 20 ng/mL induced a higher IgG production than concentrations of 100 ng/mL. Our patient presented lupus flares with PRL levels between 20 and 40 ng/mL and had remission of symptoms with PRL concentrations above 100 ng/mL (Figure 1). On the other hand, a confounder effect of dopamine agonists is unlikely, although thank to treatment the levels of PRL decreased. Nevertheless, as we discuss below, dopamine agonists may induce Raynaud's phenomenon, but neither the plethora of other symptoms nor the development of SLE and SS observed in our patient.

On the other hand, it has been described that individuals with hyperprolactinemia may exhibit PolyA phenomena (ie, more than one AD in a single patient).^6^ Some reports have shown the association of PRL with several ADs, such as SS, SLE, vasculitis.^6^, ^7^ This is in line with our patient since she developed PolyA (ie, SLE and SS), thus suggesting a possible role of PRL in this phenomenon. Although the mechanism is not clear, it was described that HLA‐DRB1 alleles (*04:01,*03:01) and microsatellite marker alleles close to the PRL gene (D6S422 and D6S285 in chromosome 6) in women with RA and SLE were associated through linkage disequilibrium with ADs.^8^ This differs from our patient who carried HLA‐DRB1*10:01. Nevertheless, some polymorphisms, such as HLA‐DRB1*10:01 in RA,^9^ HLA‐DRB1*15:01 in multiple sclerosis (MS),^10^ and HLA‐DRB1*15:03 in SLE,^11^ were found in this patient, thus suggesting that PRL in subjects with genetic predisposition may trigger the development of autoimmunity. Further analysis is warranted to clarify the role of novel HLA alleles, which in association with hyperprolactinemia may induce the development of ADs.

In addition, as shown in the pedigree, the patient exhibited a high genetic burden, set by the existence of ADs in first relatives. This phenomenon is known as “familial autoimmunity”,^12^ which has been previously associated with the development of PolyA.^13^ SLE, RA, and SS were previously diagnosed in her first‐degree relatives (Figure 2), which could be associated with a higher risk of developing PolyA. Interestingly, a relative also developed microadenoma in association with ADs. Lauruche et al^14^ found that patients with functioning and non‐functioning pituitary adenomas showed a high frequency of ADs, including autoimmune thyroid disease, Addison's disease, MS, and SS. Although the mechanisms associated with this phenomena are not clear, some families exhibit a high frequency of pituitary adenomas, and more recently, the presence of familial prolactinoma was associated with genes, such as the aryl hydrocarbon receptor interacting protein (*AIP*) gene,^15^ which in turn is related to inflammatory conditions.^16^ These data advocate for the key role of genetics in the development of pituitary adenomas, and the association between PRL and PolyA based on genetic background.

Treatment effects with dopamine agonists are wide and variable.^17^ As of 2 April 2018, seven cases of Raynaud's phenomenon during cabergoline treatment were reported to the Food and Drug Administration; interestingly, all the reports were within 5 to 10 years of cabergoline use (Cabergoline and Raynaud's phenomenon ‐from FDA reports—https://www.ehealthme.com/ds/cabergoline/raynaud-s-phenomenon/-accessed 2 April 2018). Further, some studies suggested that activation of dopamine receptors (type 1 and type 2) could increase production of IL‐6 and IL‐17.^18^, ^19^ These cytokines are strongly associated with the development of ADs, including RA, SLE, and SS.^20^, ^21^, ^22^, ^23^ Nevertheless, an Iranian pilot randomized double‐blind clinical trial assessed the effect of cabergoline in RA patients, showing improvement in tender and swollen joint count, and patients pain assessment. The authors suggest that these findings support the utility of dopamine agonist in RA management.^24^ These results differ from our patient, since she developed musculoskeletal symptoms after PRL levels decreased associated with the treatment with cabergoline. We hypothesize that administration of dopamine agonists in patients with a permissive genetic background may act as adjuvants triggering autoimmunity.

Finally, pituitary gland size frequently decreases after treatment with dopamine agonists.^25^ Size reduction is expected to be symmetric^26^; however, asymmetry of pituitary gland in STNMR following cabergoline treatment was observed in this patient (Figure 3B). This phenomenon was previously reported,^27^ and it may be associated with treatment effectiveness. Although there was not a prior comparative NMR, we consider that the presence of this pituitary asymmetry was associated with imagenological response to treatment.

## 4 CONCLUSION

In conclusion, PRL levels showed relationship with autoimmunity and could be associated with PolyA development in susceptible individuals. It appears to act as a disease activity biomarker for ADs in patients with PolyA, especially at levels between 20 and 40 ng/mL. Patients with ADs, especially with PolyA, should be tested for hyperprolactinemia, and additional studies aiming to clarify the influence of slightly raised levels of PRL in activity and severity of disease are warranted.

## CONFLICT OF INTEREST

The authors have no conflicts of interest to declare.

## AUTHOR CONTRIBUTION

MR and JMA: conducted the clinical examination and analysis of clinical records, JMA, MR, YR, and CRS: performed the literature research and wrote the manuscript. All authors approved the final version of the manuscript.

## CONSENT

Written consent was obtained from the patient prior to writing this case report.
